# Sparse multiple co-Inertia analysis with application to integrative analysis of multi -Omics data

**DOI:** 10.1186/s12859-020-3455-4

**Published:** 2020-04-15

**Authors:** Eun Jeong Min, Qi Long

**Affiliations:** Department of Biostatistics, Epidemiology and Informatics, University of Pennsylvania, 423 Guardian Dr, Philadelphia, 19104 USA

**Keywords:** Multiple co-inertia analysis, *l*_0_ penalty, Network penalty, Structural information, Gene network information, Integrative analysis, High-dimensional data, -omics data

## Abstract

**Background:**

Multiple co-inertia analysis (mCIA) is a multivariate analysis method that can assess relationships and trends in multiple datasets. Recently it has been used for integrative analysis of multiple high-dimensional -omics datasets. However, its estimated loading vectors are non-sparse, which presents challenges for identifying important features and interpreting analysis results. We propose two new mCIA methods: 1) a sparse mCIA method that produces sparse loading estimates and 2) a structured sparse mCIA method that further enables incorporation of structural information among variables such as those from functional genomics.

**Results:**

Our extensive simulation studies demonstrate the superior performance of the sparse mCIA and structured sparse mCIA methods compared to the existing mCIA in terms of feature selection and estimation accuracy. Application to the integrative analysis of transcriptomics data and proteomics data from a cancer study identified biomarkers that are suggested in the literature related with cancer disease.

**Conclusion:**

Proposed sparse mCIA achieves simultaneous model estimation and feature selection and yields analysis results that are more interpretable than the existing mCIA. Furthermore, proposed structured sparse mCIA can effectively incorporate prior network information among genes, resulting in improved feature selection and enhanced interpretability.

## Background

Large scale -omics studies have become common partly as a result of rapid advances in technologies. Many of them generate multiple -omics datasets on the same set of subjects. For example, cancer studies generate datasets using the NCI-60 cell line panel, a group of 60 human cancer cell lines used by the National Cancer Institute (NCI). Various types of -omics datasets such as gene expression or protein abundance from this cell line panel are generated and available via a web application CellMiner [32]. Another example can be found at The Cancer Genome Atlas (TCGA) repository that contains multiple types of -omics datasets such as genotype, mRNA, microRNA, and protein abundance data collected from the same set of subjects. The abundance of such datasets has created increasing needs in advanced methods for integrative analysis beyond separated analyses. Integrative analysis enables us not only to understand underlying relationships among multiple datasets but also discover more biologically meaningful results that may not be found from analysis of a single dataset. As a response to increasing needs, there have been continuous efforts in developing such methods.

Tenenhaus and Tenenhaus [36] reviewed various methods for integrative analysis of multiple datasets from the same set of subjects. Canonical correlation analysis [17] is one popular method for integrative analysis of two datasets measured on the same set of subjects. For each of two datasets, CCA seeks a linear transformation so that correlation between two transformed datasets is maximized. It is a prototype method to use a correlation-based objective function. Based on CCA, various extended methods have been proposed to integrate more than two datasets into a single model. Some examples are [4, 16, 41], [12, 12], and [15].

Covariance-based criteria is another way to construct an objective function. Tucker’s inner-battery factor analysis [38] is the seminal paper for investigating covariance structures between two datasets. Various approaches have been proposed to extend the method to an integrative model for more than two datasets. [39], [13, 19], and [8] are some examples.

Multiple co-inertia analysis [6] is another integrative analysis method employing a covariance-based objective function to identify common relationships and assess concordance among multiple datasets. This method finds a set of loading vectors for multiple *K*≥2 datasets and a so-called “synthetic" center of all datasets such that a sum of squared covariances between each of linearly transformed datasets and a synthetic center is maximized. Recently it has been applied to an integrative analysis of multiple -omics datasets [24]. However, estimated loading vectors of mCIA are nonsparse. That is, if we want to apply mCIA for analyzing two gene expression data, every gene in each data has nonzero coefficient, making it difficult to interpret the results. This has been noted as a weakness of the method [20, 25]. In statistical literature, sparse estimation has been suggested as a remedy for this type of problem and has shown good performance in genomics or biological data [22, 34].

In this paper, we propose a novel approach that imposes a sparsity constraint on mCIA method, sparse mCIA (smCIA). This model conducts estimation and variable selection simultaneously. Non-sparsity poses significant challenges not only in developing an accurate model, but also in interpreting the results. Ultra-high dimensionality is the inherited nature of -omics datasets, thus statistical models for analyzing -omics datasets benefit from feature selection procedure. To address this issue, it is desirable to employ a sparsity in the model. However, it has not been introduced in the mCIA framework to the best of our knowledge. The regularized generalized CCA framework [37] encompasses many integrative methods including mCIA and a sparse version of generalized CCA as its special cases, but it does not include a sparsity-constrained mCIA as its special case.

Also, we propose to extend smCIA, structured sparse mCIA (ssmCIA) that incorporates the structural information among variables to guide the model for obtaining more biologically meaningful results. It is well-known that gene expressions are controlled by the gene regulatory network (GRN) [31]. Incorporation of those known prior structural knowledge among genes is one of potential approaches to improve analysis results. There are continuing interests in developing statistical methods toward this direction [21, 26, 27]. To incorporate structural knowledge, we employ another penalty term in the objective function of smCIA so that we can guide the model to achieve the improved feature selection.

## Methods

Before introducing two proposed models, we briefly review the classical mCIA problem.

Suppose that we have *K* datasets from *n* subjects, i.e., *K* data triplets $(\boldsymbol {X}_{k}, \boldsymbol {D}, \boldsymbol {Q}_{k})_{k=1}^{K}, \boldsymbol {X}_{k} \in \mathbb {R}^{n \times p_{k}}, \boldsymbol {D} \in \mathbb {R}^{n \times n}, \boldsymbol {Q}_{k} \in \mathbb {R}^{p_{k} \times p_{k}}$, and ***w***=(*w*_1_,…,*w*_*K*_) for *k*=1,…,*K*. ***D*** is a diagonal weight metric of the space $\mathbb {R}^{n}, \boldsymbol {Q}_{k}$ is a diagonal weight metric of the space $\mathbb {R}^{p_{k}}$, and *w*_*k*_ is a positive weight for the *k*-th dataset such that $\sum w_{k} = 1$. Without loss of generality, assume that ***X***_*k*_ is column-wise centered and standardized.

There are various ways to construct ***D***. The simplest way is to use the identity matrix for ***D***, equal weights for each sample. Or, it can be used to put strong emphasis on some reliable samples compared to other samples by putting higher weights. Also possible sampling bias or duplicated observations can be adjusted via constructing appropriate ***D*** matrix. In specific, we can estimate the probability of selection for each individual in the sample using available covariates in the dataset and use the inverse of the estimated probability as a weight of each individual for adjustment. Later in our real data analysis, we use the identity matrix for ***D***.

For ***Q***_*k*_, we use the proportions defined as the column sums divided by the total sum of the absolute values of the *k*-th dataset, following the similar approaches used in the literature [7, 9, 24, 25]. In this way, we put higher weights on the genes with higher variability. Or, we can construct ***Q*** matrices such that some genes known to be associated with a clinical phenotype of interest have higher weights. Also, it would be another possible approaches to construct ***Q*** based on functional annotation following recent methods, originally proposed for a rare variant test for an integrative analysis [3, 14].

### Multiple co-Inertia analysis (mCIA)

The goal of mCIA is to find a set of vectors $\boldsymbol {u}_{k} \in \mathbb {R}^{p_{k}}, k=1,\ldots,K$, and a vector $\boldsymbol {v} \in \mathbb {R}^{n}$, such that the weighted sum of $(\boldsymbol {v} \intercal \boldsymbol {D} \boldsymbol {X}_{k} \boldsymbol {Q}_{k} \boldsymbol {u}_{k})^{2}$ is maximized. The objective function of mCIA problem is defined as follows,

1 $$\begin{array}{*{20}l} & \max_{\boldsymbol{v}, \boldsymbol{u}_{1},\ldots,\boldsymbol{u}_{K}} \sum^{K}_{k=1} w_{k} (\boldsymbol{v}^{\intercal} \boldsymbol{D} \boldsymbol{X}_{k} \boldsymbol{Q}_{k} \boldsymbol{u}_{k})^{2} \\ & \text{s.t} \quad \boldsymbol{u}_{k}^{\intercal} \boldsymbol{Q}_{k} \boldsymbol{u}_{k} = 1, k=1,\ldots,K, \quad \boldsymbol{v}^{\intercal} \boldsymbol{D} \boldsymbol{v} = 1,  \end{array} $$

where (***u***_1_,…,***u***_*K*_) denotes a set of co-inertia loadings (or coefficients) and ***v*** is a synthetic center [24]. The synthetic center ***v*** can be understood as a reference structure in the sample space. Loading vectors (***u***_1_,…,***u***_*K*_) are the set of coefficients that maximizes the objective function.

It has been shown that the vector ***v*** of problem (1) can be found by solving the following eigenvalue problem [6],

$$\boldsymbol{X}^{\dagger} \boldsymbol{Q}^{\dagger} {\boldsymbol{X}^{\intercal\dagger}} \boldsymbol{D} \boldsymbol{v} = \lambda \boldsymbol{v}, $$

where $\boldsymbol {X}^{\dagger } = \left [w_{1}^{1/2}\boldsymbol {X}_{1}, w_{2}^{1/2}\boldsymbol {X}_{2}, \ldots, w_{K}^{1/2}\boldsymbol {X}_{K}\right ] \in \mathbb {R}^{n \times \sum p_{k}}$ is the merged table of *K* weighted datasets and $\boldsymbol {Q}^{\dagger } \in \mathbb {R}^{\sum p_{k} \times \sum p_{k}}$ is the matrix that has ***Q***_1_,…,***Q***_*K*_ as its diagonal blocks. Given the reference vector ***v*** defined above, the loading vectors ***u***_*k*_,*k*=1,…,*K* are obtained by $\boldsymbol {u}_{k} = \boldsymbol {X}_{k}^{\intercal } \boldsymbol {D} \boldsymbol {v} / \| \boldsymbol {X}_{k}^{\intercal } \boldsymbol {D} \boldsymbol {v} \|_{\boldsymbol {Q}_{k}}$.

The second set of loadings orthogonal to the first set can be obtained by repeating the above procedure to the residual datasets calculated using a deflation method [10, Chap7.1.2].

We propose a new mCIA approach that enforces sparsity on the set of loading vectors for all datasets. Consider the following problem, which is another representation of (1),

2 $$ \begin{aligned} \underset{{\boldsymbol{b}, \boldsymbol{a}_{1},\ldots, \boldsymbol{a}_{K}}}{maximize} & \quad \sum^{K}_{k=1} \left(\boldsymbol{b}^{\intercal} \tilde{{\boldsymbol{X}}_{k}} \boldsymbol{a}_{k}\right)^{2}, & \text{s.t} \; \boldsymbol{a}_{k}^{\intercal}\boldsymbol{a}_{k} = 1, \; \boldsymbol{b}^{\intercal}\boldsymbol{b} = 1. \end{aligned}   $$

where $\tilde {\boldsymbol {X}}_{k} = \sqrt {w_{k}} \boldsymbol {D}^{1/2} \boldsymbol {X}_{k} \boldsymbol {Q}_{k}^{1/2} \in \mathbb {R}^{n\times p_{k}}, \boldsymbol {a}_{k} = \boldsymbol {Q}_{k}^{1/2}\boldsymbol {u}_{k} \in \mathbb {R}^{p_{k}}$, and $\boldsymbol {b} = \boldsymbol {D}^{1/2} \boldsymbol {v} \in \mathbb {R}^{n}$. The problem (2) is a multi-convex problem, which is a convex problem with respect to ***a***_*k*_ while others $\phantom {\dot {i}\!}\boldsymbol {a}_{k'},k'=1,\ldots,k-1,k+1, \ldots,K$ and ***v*** are fixed. This enables us to apply an iterative algorithm for finding a solution set (***b***,***a***_1_,…,***a***_*K*_).

First, for fixed ***a***_*k*_,*k*=1,…,*K*, the problem (2) becomes

3 $$ \begin{aligned} \underset{\boldsymbol{b}}{maximize} & \quad \sum^{K}_{k=1} \left(\boldsymbol{b}^{\intercal} \tilde{\boldsymbol{X}}_{k} \boldsymbol{a}_{k}\right)^{2},& \text{s.t} \quad \boldsymbol{b}^{\intercal}\boldsymbol{b} = 1. \end{aligned}  $$

where the objective function is convex with respect to ***b***. Indeed, above problem can be optimized via Eigenvalue decomposition. Consider the Lagrangian formulation of (3), $L(\boldsymbol {b}) = \sum ^{K}_{k=1} \left (\boldsymbol {b}^{\intercal } \tilde {\boldsymbol {X}}_{k} \boldsymbol {a}_{k}\right)^{2} - \lambda (\boldsymbol {b}^{\intercal } \boldsymbol {b} -1)$, where *λ* is a Lagrangian multiplier. To obtain a solution, we take a derivative of *L* with respect to ***b*** and solve the equation by setting the derivative equal to zero as follows, $ \frac {\partial L}{\partial \boldsymbol {b}} = 2 \sum ^{K}_{k=1} \left (\boldsymbol {b}^{\intercal } \tilde {\boldsymbol {X}}_{k} \boldsymbol {a}_{k}\right) \tilde {\boldsymbol {X}}_{k} \boldsymbol {a}_{k} - 2\lambda \boldsymbol {b} = 2 \left (\sum ^{K}_{k=1} \boldsymbol {M}_{k} \boldsymbol {b} - \lambda \boldsymbol {b}\right) = 0,$ where $\boldsymbol {M}_{k} = \tilde {\boldsymbol {X}}_{k} \boldsymbol {a}_{k} \boldsymbol {a}_{k}^{\intercal } \tilde {\boldsymbol {X}}_{k}^{\intercal } \in  {n \times n}$. The optimal ***b*** is the first eigenvector of $\sum _{k=1}^{K} \boldsymbol {M}_{k}$.

As a next step for finding a solution of ***a***_1_, we fix ***b*** and ***a***_*k*_,*k*=2,…,*K*. Then we have

4 $$ \begin{aligned} \underset{\boldsymbol{a}_{1}}{maximize} & \quad \boldsymbol{a}_{1}^{\intercal} \boldsymbol{N}_{1} \boldsymbol{a}_{1}, & \text{s.t} \quad \boldsymbol{a}_{1}^{\intercal} \boldsymbol{a}_{1} =1, \end{aligned}   $$

where $\boldsymbol {N}_{1} = \tilde {\boldsymbol {X}}_{1}^{\intercal } \boldsymbol {b} \boldsymbol {b}^{\intercal } \tilde {\boldsymbol {X}}_{1}$. Notice that the problem (4) is the eigenvalue decomposition problem. The first eigenvector of ***N***_1_ is the optimal ***a***_1_ and the corresponding eigenvalue is the maximized objective value at the optimal value of ***a***_1_. Rest of loading vectors ***a***_2_,…,***a***_*K*_ can be estimated by applying the same procedure as ***a***_1_. From the set of estimated vectors $(\hat {\boldsymbol {b}}, \hat {\boldsymbol {a}_{1}},\ldots,\hat {\boldsymbol {a}_{K}})$, we recover a solution of the original mCIA, $(\hat {\boldsymbol {v}}, \hat {\boldsymbol {u}_{1}},\ldots,\hat {\boldsymbol {u}_{K}})$, by premultiplying $\boldsymbol {D}^{-1/2}, \boldsymbol {Q}^{-1/2}_{1},\ldots, \boldsymbol {Q}^{-1/2}_{K}$ to $(\hat {\boldsymbol {b}}, \hat {\boldsymbol {a}_{1}}, \ldots, \hat {\boldsymbol {a}_{K}})$ respectively.

The subsequent sets of vectors $\left (\boldsymbol {v}^{(r)}, \boldsymbol {u}^{(r)}_{1},\ldots, \boldsymbol {u}^{(r)}_{K}\right), r=2,\ldots,\min (n, p_{1}, \ldots, p_{K})$ which are orthogonal to all sets of previously estimated vectors can be estimated by applying the same procedure to the residual data matrices $\boldsymbol {X}^{(r)}_{1}, \ldots, \boldsymbol {X}^{(r)}_{K}$ with respect to the previously estimated vectors $\left (\boldsymbol {v}^{(r')}, \boldsymbol {u}_{1}^{(r')}, \ldots,\boldsymbol {u}_{K}^{(r')}\right), r'=1,\ldots, r-1$ using a deflation technique.

### Sparse mCIA

For obtaining interpretable results, sparsity on coefficient loading vectors (***a***_1_,…,***a***_*K*_) is desirable. To this end, we will impose a sparsity constraint on the transformed loading vectors ***a***_1_,…,***a***_*K*_. Note that we do not put a sparsity constraint on the reference vector ***b*** in the sample space. Sparsity on (***a***_1_,…,***a***_*K*_) can be transferred to the original loading vectors (***u***_1_,…,***u***_*K*_) because the weight matrices ***Q***_1_,…,***Q***_*K*_ are assumed to be diagonal matrices.

Given ***b*** and ***a***_*k*_,*k*=2,…,*K*, we propose to add the *l*_0_-sparsity constraint to (4) for obtaining a sparse estimate of ***a***_1_ as follows,

5 $$ \begin{aligned} \underset{\boldsymbol{a}_{1}}{maximize} & \quad \boldsymbol{a}_{1}^{\intercal} \boldsymbol{N}_{1} \boldsymbol{a}_{1},& \text{s.t} \; \boldsymbol{a}_{1}^{\intercal} \boldsymbol{a}_{1} = 1,\; \| \boldsymbol{a}_{1} \|_{0} \leq s_{1}, \end{aligned}   $$

where $\boldsymbol {N}_{1} = \tilde {\boldsymbol {X}}_{1}^{\intercal } \boldsymbol {b} \boldsymbol {b}^{\intercal } \tilde {\boldsymbol {X}}_{1}$ and *s*_1_ is a pre-defined positive integer value less than *p*_1_.

To tackle our problem (5), we will utilize the algorithm recently proposed by [35]. They proposed the truncated Rayleigh flow method (Rifle), which solves the maximization problem of the *l*_0_-sparsity constrained generalized Rayleigh quotient. It is well known that the optimization problem of the generalized Rayleigh quotient with respect to $\boldsymbol {\omega }\in \mathbb {R}^{p}$,

6 $$ f(\boldsymbol{\omega}) = \boldsymbol{\omega}^{\intercal} \boldsymbol{R}_{1} \boldsymbol{\omega} / \boldsymbol{\omega}^{\intercal} \boldsymbol{R}_{2} \boldsymbol{\omega},   $$

where $\boldsymbol {R}_{1},\boldsymbol {R}_{2}\in \mathbb {R}^{p\times p}$ are symmetric real-valued matrices, is same as the generalized eigenvalue problem. Our objective criterion is a specific case of the generalized eigenvalue problem with ***R***_1_=***N***_1_ and $\phantom {\dot {i}\!}\boldsymbol {R}_{2} = \boldsymbol {I}_{p_{1}}$, which allows us to use Rifle for solving our problem. The algorithm is a simple iterative procedure consisting of the gradient descent algorithm and hard-thresholding steps. At each iteration, the most biggest *s*_1_ elements of the solution from the gradient descent step are left as nonzero and others are forced to be zero. The same procedure is applied for estimating remaining loading vectors ***a***_2_,…,***a***_*K*_. The complete pseudo-algorithm of smCIA problem is summarized in Algorithm 1.

### Structured sparse mCIA

We propose another new model that incorporates prior known network information among features. To this end, we employ the Laplacian penalty on the sparse mCIA model to obtain more biologically meaningful results.

Let ${\mathcal {G}}_{1}=\{\boldsymbol {C}_{1},\boldsymbol {E}_{1}, \boldsymbol {W}_{1}\}$ denote a weighted and undirected graph of variables in ***X***_1_, where ***C***_1_ is the set of vertices corresponding to the *p*_1_ features (or nodes), ***E***_1_={*i*∼*j*} is the set of edges that connect features *i* and *j*, and ***W***_1_ contains the weights for all nodes. Given $\mathcal {G}_{1}=\{\boldsymbol {C}_{1},\boldsymbol {E}_{1}, \boldsymbol {W}_{1} \}$, the (*i*,*j*)-th element of the normalized Laplacian matrix ***L***_1_ of ***X***_1_ is defined by

$$\begin{aligned} \boldsymbol{L}_{1}(i,j) = \left\{ \begin{array}{ll} 1-w_{1}(i,j)/d_{i}, & \text{if }i=j \text{ and} \ d_{i}\neq 0, \\ -w_{1}(i,j)/\sqrt{d_{i} d_{j}}, & \text{if}\ i \text{ and}\ j \text{ are adjacent}, \\ 0, & \text{otherwise,} \end{array} \right. \end{aligned} $$

where *w*_1_(*i*,*j*) is a weight of the edge *e*=(*i*∼*j*) and *d*_*i*_ is a degree of the vertex *i* defined as $\sum _{i\sim j} w_{1}(i,j)$. It is easily shown that $p(\boldsymbol {u}_{1}; \boldsymbol {L}_{1})= \boldsymbol {u}_{1}^{\intercal } \boldsymbol {L}_{1} \boldsymbol {u}_{1}$ becomes zero if the prior known network information of ***L***_1_ agrees with the true network existing among ***X***_1_.

For fixed ***b*** and ***a***_*k*_,*k*=2,…,*K*, consider the following optimization problem,

7 $$ \begin{aligned} \underset{\boldsymbol{a}_{1}}{maximize} & \quad \boldsymbol{a}_{1}^{\intercal} \boldsymbol{N}_{1} \boldsymbol{a}_{1} - \lambda_{1} \boldsymbol{a}_{1}^{\intercal} \tilde{\boldsymbol{L}}_{1} \boldsymbol{a}_{1}\\ \text{s.t} & \quad \boldsymbol{a}_{1}^{\intercal} \boldsymbol{a}_{1} = 1, \quad \| \boldsymbol{a}_{1} \|_{0} \leq s_{1},\\ \end{aligned}   $$

where $\boldsymbol {N}_{1} = \tilde {\boldsymbol {X}}_{1}^{\intercal } \boldsymbol {b} \boldsymbol {b}^{\intercal } \tilde {\boldsymbol {X}}_{1}, s_{1}$ is a pre-defined positive integer value less than *p*_1_,*λ*_1_ is a pre-defined network penalty parameter, and $\tilde {\boldsymbol {L}}_{1} = \boldsymbol {Q}_{1}^{-1/2} \boldsymbol {L}_{1} \boldsymbol {Q}_{1}^{-1/2}$ is a transformed Laplacian matrix that contains the network information among variables of ***X***_1_. To solve (7), the network penalty needs to be minimized, which implies that the penalty encourages the model to estimate ***a***_1_ to be in agreement with the incorporated network information contained in the $\tilde {\boldsymbol {L}}_{1}$.

We again employ Rifle for solving (7). The objective function of (7) become $\boldsymbol {a}_{1}^{\intercal } \boldsymbol {R}_{1} \boldsymbol {a}_{1}$ where $\boldsymbol {R}_{1} = \boldsymbol {N}_{1} - \lambda _{1} \tilde {\boldsymbol {L}}_{1}$. Rifle requires ***R***_1_ to be symmetric and $\boldsymbol {N}_{1} - \lambda _{1} \tilde {\boldsymbol {L}}_{1}$ satisfies the condition since both ***N***_1_ and $\tilde {\boldsymbol {L}}_{1}$ are symmetric. Like smCIA algorithm, the estimation of remaining loading vectors ***a***_2_,…,***a***_*K*_ is same as that of ***a***_1_. The complete pseudo-algorithm of ssmCIA problem is summarized in Algorithm 1.


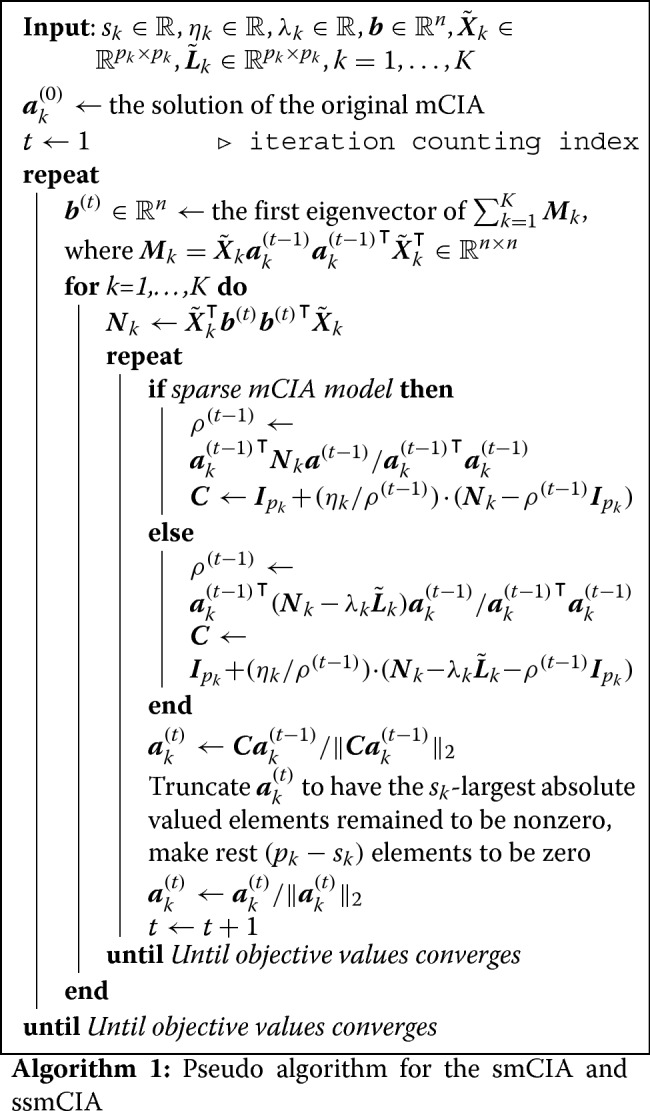


### Choice of tuning parameters

In our methods, we have *K* and 2*K* parameters required to be tuned for smCIA and ssmCIA, respectively. Denote the set of tuning parameters as

$$\begin{aligned} \boldsymbol{\lambda} = \left\{ \begin{array}{ll} \{s_{k}, k=1,\ldots,K\}, & \text{if smCIA,}\\ \{s_{k}, \lambda_{k}, k=1,\ldots,K\}, & \text{if ssmCIA}. \end{array} \right. \end{aligned} $$

We employ a *T*-fold cross validation (CV) method to select the best tuning parameter set. We set the range of grid points for each parameters from several initial trials. We divide each dataset into *T* subgroups and calculate the CV objective value defined as follows,

$$ CV(\boldsymbol{\lambda}) = \frac{ (T-1) \sum_{k=1}^{K} \sum_{t=1}^{T} cv_{t,k} }{ T \sum_{k=1}^{K} \sum_{t=1}^{T} \left(cv_{t,k} - \sum_{k=1}^{K} \sum_{t=1}^{T} cv_{t,k} \right)^{2} } $$

where $cv_{t,k} =\left ((\hat {\boldsymbol {b}}^{-t}(\boldsymbol {\lambda }))^{\intercal } \tilde {\boldsymbol {X}}^{t}_{k} \hat {\boldsymbol {a}}_{k}^{-t}(\boldsymbol {\lambda }) \right)^{2}$, and $\hat {\boldsymbol {a}}_{k}^{-t}(\boldsymbol {\lambda })$ and $\hat {\boldsymbol {b}}^{-t}(\boldsymbol {\lambda }), t=1,\ldots,T$ are estimated loading vectors and reference vectors from the training data $\tilde {\boldsymbol {X}}_{k}^{-t}$ using a tuning parameter set ***λ***. This can be considered as a scaled version of the CV objective value used in [40]. Unlike CCA whose correlation values are always within a range [−1,1], co-inertia values are not limited to be within a certain range. We overcome this problem by standardizing all co-inertia values used for the cross validation.

There is another set of parameters in the algorithm, the stepsize *η*_*k*_ of the gradient descent step. [35] suggests that *η*_*k*_<1/maximum eigenvalue of ***R***_2_, where ***R***_2_ is the matrix in the denominator of the Rayleigh function (6). Since ***R***_2_ is the identity matrix in smCIA and ssmCIA problem, the maximum value of *η*_*k*_ is 1. We also tune this value by exploring multiple values within (0,1] and select the best value using the cross validation.

Lastly, we use the nonsparse solution of (***u***_1_,…,***u***_*k*_,***v***) from mCIA as a starting point.

## Simulation study

### Synthetic data generation

We use a latent variable model to generate synthetic *K* datasets related to each other. Let *θ* be a latent variable such that *θ*∼*N*(0,*σ*^2^) and it affects to *K* sets of random variables $\boldsymbol {x}_{k} = \theta \boldsymbol {a}_{k}^{\intercal } + \boldsymbol {\epsilon }_{k}^{\intercal } \in \mathbb {R}^{p_{k} \time 1}, k=1,\ldots, K$, where $\phantom {\dot {i}\!}\boldsymbol {\epsilon }_{k} \sim N(\boldsymbol {0}_{p_{k}}, \boldsymbol {\Sigma }_{k})$ and ***a***_*k*_ is set to be same as the first eigenvector of the matrix ***Σ***_*k*_. Then following calculation

$${}\begin{aligned} \mathrm{E}(\boldsymbol{X}_{k}^{\intercal} \boldsymbol{b} \boldsymbol{b}^{\intercal} \boldsymbol{X}_{k}) & = \sum_{i}^{n} b_{i}^{2} \mathrm{E} [ \left(\theta_{i} \boldsymbol{a}_{k} + \boldsymbol{\epsilon}_{k,i}\right)\left(\theta_{i} \boldsymbol{a}_{k}^{\intercal}+ \boldsymbol{\epsilon}_{k,i}^{\intercal}\right) ]\\ & = \mathrm{E}\left[\theta^{2} \boldsymbol{a}_{k} \boldsymbol{a}_{k}^{\intercal} + \theta \boldsymbol{a}_{k} \boldsymbol{\epsilon}_{k,i}^{\intercal} + \theta \boldsymbol{\epsilon}_{k}\boldsymbol{a}_{k}^{\intercal} + \boldsymbol{\epsilon}_{k}\boldsymbol{\epsilon}_{k}^{\intercal}\right]\\ & = \sigma^{2} \boldsymbol{a}_{k} \boldsymbol{a}_{k}^{\intercal} + \boldsymbol{\Sigma}_{k}\\ & = \left(\sigma^{2} + \gamma_{1}\right) \boldsymbol{a}_{k} \boldsymbol{a}_{k}^{\intercal} + \sum_{j=2}^{p_{k}}\gamma_{j} \boldsymbol{e}_{j} \boldsymbol{e}_{j}^{\intercal} \end{aligned} $$

verifies that ***a***_*k*_ is same as ***e***_1_, the first eigenvector of the matrix $\mathrm {E}\left (\boldsymbol {X}_{k}^{\intercal } \boldsymbol {b} \boldsymbol {b}^{\intercal } \boldsymbol {X}_{k}\right)$ with the corresponding eigenvalue *nσ*^2^+*γ*_1_, where (*γ*_*j*_,***e***_*j*_),*j*=1,…,*p*_*k*_ are eigen-pairs of ***Σ***_*k*_.

Following calculation is for cross-covariance matrices in the model.

$$\begin{aligned} \mathrm{E}(\boldsymbol{X}_{l}^{\intercal} \boldsymbol{b} \boldsymbol{b}^{\intercal} \boldsymbol{X}_{m}) & = \sum_{i}^{n} b_{i}^{2} \mathrm{E} \left[ (\theta_{i} \boldsymbol{a}_{l} + \boldsymbol{\epsilon}_{l,i})\left(\theta_{i} \boldsymbol{a}_{m}^{\intercal}+ \boldsymbol{\epsilon}_{m,i}^{\intercal}\right) \right]\\ & = \mathrm{E}\left[\theta^{2} \boldsymbol{a}_{l} \boldsymbol{a}_{m}^{\intercal} + \theta \boldsymbol{a}_{l} \boldsymbol{\epsilon}_{m}^{\intercal} + \theta \boldsymbol{\epsilon}_{l}\boldsymbol{a}_{m}^{\intercal} + \boldsymbol{\epsilon}_{l}\boldsymbol{\epsilon}_{m}^{\intercal}\right]\\ & = \sigma^{2} \boldsymbol{a}_{l} \boldsymbol{a}_{m}^{\intercal}.\\ \end{aligned} $$

Our complete generative model simulates a concatenated dataset $\boldsymbol {X}^{\intercal } = \left [\boldsymbol {X}_{1}^{\intercal } \, \boldsymbol {X}_{2}^{\intercal } \, \cdots \, \boldsymbol {X}_{K}^{\intercal }\right ] \in \mathbb {R}^{\sum p_{k} \times n}$ from the normal distribution with the mean $\boldsymbol {0}_{\sum p_{k}}$ and the variance $\Sigma _{T} \in \mathbb {R}^{\sum p_{k} \times \sum p_{k}}$, where

$$ \Sigma_{T} = \begin{bmatrix} \sigma^{2} \boldsymbol{a}_{1} \boldsymbol{a}_{1}^{\intercal} + \boldsymbol{\Sigma}_{1} & \sigma^{2} \boldsymbol{a}_{1} \boldsymbol{a}_{2}^{\intercal} & \cdots & \sigma^{2} \boldsymbol{a}_{1} \boldsymbol{a}_{K}^{\intercal}\\ \sigma^{2} \boldsymbol{a}_{2} \boldsymbol{a}_{2}^{\intercal} & \sigma^{2} \boldsymbol{a}_{2} \boldsymbol{a}_{2}^{\intercal} + \boldsymbol{\Sigma}_{2} & \cdots & \sigma^{2} \boldsymbol{a}_{2} \boldsymbol{a}_{K}^{\intercal}\\ \vdots & \vdots & \vdots & \vdots \\ \sigma^{2} \boldsymbol{a}_{1} \boldsymbol{a}_{K}^{\intercal} & \sigma^{2} \boldsymbol{a}_{2} \boldsymbol{a}_{K}^{\intercal} & \cdots & \sigma^{2} \boldsymbol{a}_{K} \boldsymbol{a}_{K}^{\intercal} + \boldsymbol{\Sigma}_{K} \end{bmatrix}. $$

### Simulation design

We consider various simulation designs to compare the performance of smCIA and ssmCIA with mCIA. We compare our methods with mCIA only since the objective functions of other integrative methods such as generalized CCA or methods that have the covariance-based objective function are different from mCIA so that direct comparison is inappropriate.

We assume that there exist multiple networks among genes in each dataset, and the networks affect the relationship between datasets. We have 8 design scenarios by varying three conditions:

- *σ*^2^, the variance of the latent variable,
- *n*_*el*_, the number of elements in each network,
- *n*_*en*_, the number of effective networks among whole networks.

We generate 100 Monte Carlo (MC) datasets. For each MC dataset, we generate *n*=200 observations of each three random variables $\boldsymbol {x}_{1}\in \mathbb {R}^{300}, \boldsymbol {x}_{2} \in \mathbb {R}^{400}$, and $\boldsymbol {x}_{3}\in \mathbb {R}^{500}$. There are 5 networks among each of ***x***_1_,***x***_2_, and ***x***_3_ and 10 or 20 elements *n*_*el*_ in each network. Among *n*_*el*_ genes of each network, the first indexed gene is the main gene that are connected to all other genes within the network. This means that the first indexed gene of each network in the simulation design with *n*_*el*_=20 has the higher weight compared to the one in the simulation with *n*_*el*_=10. For the number of effective networks *n*_*en*_, we consider two cases. One case assumes that some networks affect relationships among datasets by setting *n*_*en*_=(3,4,5), while the other case assumes that all existing networks affect relationships, *n*_*en*_=(5,5,5). Also, we consider two values for *σ*^2^=(1.2,2.5), the higher *σ*^2^ value leads to the higher first eigenvalue of $\mathrm {E}\left (\boldsymbol {X}_{k}^{\intercal } \boldsymbol {b} \boldsymbol {b}^{\intercal } \boldsymbol {X}_{k}\right) $.

All true loadings make the network penalty zero. Thus we expect that ssmCIA performs better compared to smCIA since ssmCIA is encouraged to estimate the coefficient loadings minimizing the network penalty. All simulation scenarios and corresponding true coefficient loadings are summarized in Table 1. In addition, we consider incorporating incorrect network information in the first scenario to show the robustness of ssmCIA. Results of the additional simulation studies can be found in the supplementary materials.

**Table 1:** Simulation designs for each scenario and corresponding true loading vectors. All true vectors are normalized to have *l*_2_-norm 1

*n*_*en*_=(3,4,5)		
	*n*_*el*_=10	*n*_*el*_=20
*σ*^2^=1.2	scenario 1	scenario 2
	$\boldsymbol {a}_{1}=((\boldsymbol {1}_{3}, \boldsymbol {0}_{27})^{\intercal } \bigotimes \boldsymbol {1}_{10})$	$\boldsymbol {a}_{1}=((\boldsymbol {1}_{3}, \boldsymbol {0}_{27})^{\intercal } \bigotimes \boldsymbol {1}_{20})$
	$\boldsymbol {a}_{2}=((\boldsymbol {1}_{4}, \boldsymbol {0}_{36})^{\intercal } \bigotimes \boldsymbol {1}_{10})$	$\boldsymbol {a}_{2}=((\boldsymbol {1}_{4}, \boldsymbol {0}_{36})^{\intercal } \bigotimes \boldsymbol {1}_{20})$
	$\boldsymbol {a}_{3}=((\boldsymbol {1}_{5}, \boldsymbol {0}_{45})^{\intercal } \bigotimes \boldsymbol {1}_{10})$	$\boldsymbol {a}_{3}=((\boldsymbol {1}_{5}, \boldsymbol {0}_{45})^{\intercal } \bigotimes \boldsymbol {1}_{20})$
*σ*^2^=2.5	scenario 3	scenario 4
	$\boldsymbol {a}_{1}=((\boldsymbol {1}_{3}, \boldsymbol {0}_{27})^{\intercal } \bigotimes \boldsymbol {1}_{10})$	$\boldsymbol {a}_{1}=((\boldsymbol {1}_{3}, \boldsymbol {0}_{27})^{\intercal } \bigotimes \boldsymbol {1}_{20})$
	$\boldsymbol {a}_{2}=((\boldsymbol {1}_{4}, \boldsymbol {0}_{36})^{\intercal } \bigotimes \boldsymbol {1}_{10})$	$\boldsymbol {a}_{2}=((\boldsymbol {1}_{4}, \boldsymbol {0}_{36})^{\intercal } \bigotimes \boldsymbol {1}_{20})$
	$\boldsymbol {a}_{3}=((\boldsymbol {1}_{5}, \boldsymbol {0}_{45})^{\intercal } \bigotimes \boldsymbol {1}_{10})$	$\boldsymbol {a}_{3}=((\boldsymbol {1}_{5}, \boldsymbol {0}_{45})^{\intercal } \bigotimes \boldsymbol {1}_{20})$
*n*_*en*_=(5,5,5)		
	*n*_*el*_=10	*n*_*el*_=20
*σ*^2^=1.2	scenario 5	scenario 6
	$\boldsymbol {a}_{1}=((\boldsymbol {1}_{5}, \boldsymbol {0}_{25})^{\intercal } \bigotimes \boldsymbol {1}_{10})$	$\boldsymbol {a}_{1}=((\boldsymbol {1}_{5}, \boldsymbol {0}_{25})^{\intercal } \bigotimes \boldsymbol {1}_{20})$
	$\boldsymbol {a}_{2}=((\boldsymbol {1}_{5}, \boldsymbol {0}_{35})^{\intercal } \bigotimes \boldsymbol {1}_{10})$	$\boldsymbol {a}_{2}=((\boldsymbol {1}_{5}, \boldsymbol {0}_{35})^{\intercal } \bigotimes \boldsymbol {1}_{20})$
	$\boldsymbol {a}_{3}=((\boldsymbol {1}_{5}, \boldsymbol {0}_{45})^{\intercal } \bigotimes \boldsymbol {1}_{10})$	$\boldsymbol {a}_{3}=((\boldsymbol {1}_{5}, \boldsymbol {0}_{45})^{\intercal } \bigotimes \boldsymbol {1}_{20})$
*σ*^2^=2.5	scenario 7	scenario 8
	$\boldsymbol {a}_{1}=((\boldsymbol {1}_{5}, \boldsymbol {0}_{25})^{\intercal } \bigotimes \boldsymbol {1}_{10})$	$\boldsymbol {a}_{1}=((\boldsymbol {1}_{5}, \boldsymbol {0}_{25})^{\intercal } \bigotimes \boldsymbol {1}_{20})$
	$\boldsymbol {a}_{2}=((\boldsymbol {1}_{5}, \boldsymbol {0}_{35})^{\intercal } \bigotimes \boldsymbol {1}_{10})$	$\boldsymbol {a}_{2}=((\boldsymbol {1}_{5}, \boldsymbol {0}_{35})^{\intercal } \bigotimes \boldsymbol {1}_{20})$
	$\boldsymbol {a}_{3}=((\boldsymbol {1}_{5}, \boldsymbol {0}_{45})^{\intercal } \bigotimes \boldsymbol {1}_{10})$	$\boldsymbol {a}_{3}=((\boldsymbol {1}_{5}, \boldsymbol {0}_{45})^{\intercal } \bigotimes \boldsymbol {1}_{20})$

### Performance measures

To compare the feature selection performance of our methods in the simulations, we use sensitivity (SENS), specificity (SPEC), and Matthew’s correlation coefficient (MCC) defined as follows,

$$ \begin{aligned} \text{SENS} &= \frac{TP}{TP + FN}, \quad\text{SPEC} = \frac{TN}{FP + TN}\\ \text{MCC} &= \frac{TP \times TN - FP \times FN}{\sqrt{(TP+FP)(TP+FN)(TN+FP)(TN+FN)}}, \end{aligned} $$

where TP, TN, FP, and FN are true positives, true negatives, false positives, and false negatives, respectively. Also, we calculate the angle between the estimated loading vectors $\hat {\boldsymbol {a}}_{k}$ and the true loading vectors $\boldsymbol {a}^{*}_{k}, k=1,2,3$, to compare the estimation performance between our methods and mCIA. Angle is defined as $\angle (\hat {\boldsymbol {a}}_{k}) = \frac {\hat {\boldsymbol {a}}_{k}^{\intercal } \boldsymbol {a}^{*}_{k}}{\|\hat {\boldsymbol {a}}_{k}\|_{2}\times \|\boldsymbol {a}_{k}^{*}\|_{2}}$. If two vectors are exactly same, the calculated angle between those two vectors is 1.

### Simulation results

Simulation results are summarized in Table 2 and Table 3. First, the estimation performance of our proposed methods are superior compared to mCIA evidenced by calculated angle values. An angle value is close to 1 if the estimated loading vector is close to the true loading vector. The calculated angle values from our methods are closer to 1 than those from mCIA. Second, ssmCIA performs better than smCIA in feature selection. Note that, in our simulation scenarios, the true loadings are designed to follow the pre-defined network structure of the synthetic data. Thus we expect to observe better performance from ssmCIA than that from smCIA. In all scenarios, ssmCIA performs better than smCIA in all aspects, SENS, SPEC, MCC, and even for angle.

**Table 2:** Simulation results using sparse mCIA are shown. Sensitivity (Sens), Specificity (Spec), and Matthew’s correlation coefficient (MCC) for feature selection performance and Angle for estimation performance are calculated. 5-fold cross validation is used to choose the best tuning parameter combination in each method. Values within parenthesis are standard errors

sparse multiple CIA													mCIA		
	***a***_1_				***a***_2_				***a***_3_				***a***_1_	***a***_2_	***a***_3_
scen	Sens	Spec	MCC	Angle	Sens	Spec	MCC	Angle	Sens	Spec	MCC	Angle	Angle	Angle	Angle
1	0.675	0.991	0.754	0.885	0.74	0.991	0.803	0.901	0.77	0.991	0.82	0.905	0.882	0.847	0.830
	(0.285)	(0.018)	(0.161)	(0.081)	(0.205)	(0.014)	(0.102)	(0.052)	(0.155)	(0.012)	(0.071)	(0.037)	(0.025)	(0.028)	(0.025)
2	0.754	0.974	0.781	0.901	0.759	0.966	0.762	0.886	0.755	0.96	0.743	0.875	0.879	0.847	0.833
	(0.130)	(0.032)	(0.058)	(0.028)	(0.089)	(0.027)	(0.046)	(0.024)	(0.071)	(0.022)	(0.041)	(0.021)	(0.024)	(0.027)	(0.023)
3	0.711	0.996	0.794	0.904	0.776	0.996	0.846	0.924	0.813	0.996	0.87	0.933	0.933	0.915	0.897
	(0.316)	(0.012)	(0.200)	(0.095)	(0.231)	(0.009)	(0.134)	(0.066)	(0.177)	(0.007)	(0.096)	(0.047)	(0.011)	(0.011)	(0.015)
4	0.826	0.982	0.848	0.937	0.846	0.981	0.857	0.936	0.845	0.977	0.845	0.928	0.933	0.915	0.897
	(0.145)	(0.029)	(0.069)	(0.033)	(0.100)	(0.022)	(0.040)	(0.020)	(0.077)	(0.020)	(0.040)	(0.018)	(0.011)	(0.011)	(0.015)
5	0.771	0.986	0.816	0.908	0.763	0.989	0.812	0.902	0.764	0.991	0.819	0.903	0.882	0.847	0.83
	(0.162)	(0.020)	(0.079)	(0.042)	(0.159)	(0.015)	(0.077)	(0.040)	(0.157)	(0.012)	(0.074)	(0.039)	(0.024)	(0.028)	(0.025)
6	0.812	0.93	0.757	0.897	0.783	0.944	0.742	0.879	0.767	0.954	0.738	0.871	0.883	0.85	0.825
	(0.081)	(0.042)	(0.046)	(0.023)	(0.078)	(0.031)	(0.049)	(0.023)	(0.078)	(0.024)	(0.051)	(0.027)	(0.023)	(0.025)	(0.03)
7	0.839	0.99	0.873	0.941	0.836	0.993	0.875	0.938	0.837	0.994	0.878	0.939	0.933	0.912	0.9
	(0.161)	(0.017)	(0.087)	(0.043)	(0.159)	(0.013)	(0.083)	(0.041)	(0.160)	(0.010)	(0.082)	(0.042)	(0.011)	(0.014)	(0.013)
8	0.88	0.959	0.851	0.942	0.865	0.968	0.847	0.933	0.863	0.975	0.854	0.933	0.933	0.913	0.899
	(0.077)	(0.039)	(0.044)	(0.017)	(0.076)	(0.026)	(0.040)	(0.017)	(0.071)	(0.021)	(0.036)	(0.015)	(0.011)	(0.014)	(0.013)

**Table 3:** Simulation results using structured sparse mCIA are shown. Sensitivity (Sens), Specificity (Spec), and Matthews correlation coefficient (MCC) for feature selection performance and Angle for estimation performance are calculated. 5-fold cross validation is used to choose the best tuning parameter combination in each method. Values within parenthesis are standard errors

structured sparse multiple CIA													mCIA		
	***a***_1_				***a***_2_				***a***_3_				***a***_1_	***a***_2_	***a***_3_
scenario	Sens	Spec	MCC	Angle	Sens	Spec	MCC	Angle	Sens	Spec	MCC	Angle	Angle	Angle	Angle
1	0.71	0.994	0.786	0.897	0.767	0.993	0.827	0.913	0.79	0.992	0.837	0.915	0.882	0.847	0.830
	(0.284)	(0.011)	(0.166)	(0.088)	(0.204)	(0.009)	(0.106)	(0.056)	(0.154)	(0.008)	(0.073)	(0.041)	(0.025)	(0.028)	(0.025)
2	0.79	0.979	0.814	0.918	0.787	0.97	0.789	0.901	0.774	0.962	0.761	0.885	0.879	0.847	0.833
	(0.127)	(0.021)	(0.058)	(0.030)	(0.089)	(0.018)	(0.046)	(0.024)	(0.068)	(0.016)	(0.041)	(0.022)	(0.024)	(0.027)	(0.023)
3	0.748	0.995	0.816	0.915	0.807	0.996	0.863	0.934	0.838	0.996	0.884	0.941	0.933	0.915	0.897
	(0.300)	(0.010)	(0.186)	(0.092)	(0.221)	(0.008)	(0.126)	(0.064)	(0.171)	(0.006)	(0.091)	(0.047)	(0.011)	(0.011)	(0.015)
4	0.854	0.987	0.875	0.947	0.867	0.984	0.877	0.945	0.862	0.979	0.861	0.937	0.933	0.915	0.897
	(0.142)	(0.016)	(0.072)	(0.034)	(0.097)	(0.014)	(0.042)	(0.021)	(0.074)	(0.013)	(0.038)	(0.018)	(0.011)	(0.011)	(0.015)
5	0.798	0.986	0.833	0.919	0.791	0.989	0.831	0.913	0.793	0.992	0.838	0.915	0.882	0.847	0.83
	(0.162)	(0.016)	(0.075)	(0.042)	(0.162)	(0.012)	(0.076)	(0.043)	(0.160)	(0.009)	(0.073)	(0.042)	(0.024)	(0.028)	(0.025)
6	0.83	0.939	0.781	0.911	0.803	0.951	0.768	0.893	0.785	0.959	0.76	0.884	0.883	0.85	0.825
	(0.069)	(0.029)	(0.042)	(0.020)	(0.069)	(0.020)	(0.043)	(0.021)	(0.065)	(0.017)	(0.043)	(0.024)	(0.023)	(0.025)	(0.03)
7	0.852	0.993	0.887	0.947	0.848	0.994	0.886	0.944	0.849	0.996	0.89	0.945	0.933	0.912	0.9
	(0.158)	(0.011)	(0.087)	(0.044)	(0.157)	(0.008)	(0.083)	(0.043)	(0.156)	(0.006)	(0.081)	(0.043)	(0.011)	(0.014)	(0.013)
8	0.873	0.968	0.859	0.945	0.861	0.975	0.857	0.938	0.86	0.981	0.864	0.937	0.933	0.913	0.899
	(0.076)	(0.025)	(0.039)	(0.018)	(0.077)	(0.017)	(0.039)	(0.018)	(0.072)	(0.014)	(0.035)	(0.016)	(0.011)	(0.014)	(0.013)

Also, we have several observations by comparing the results of different scenarios, driven by the nature of our generative model. First, the performance of the methods is better in the scenarios 3(4,7,8) than the one in the scenarios 1(2,5,6) (respectively). This observation agrees with our expectation originated from the nature of our generative model. In particular, the bigger *σ*^2^ makes the first eigenvalue of the matrix $\boldsymbol {X}_{k}^{\intercal } \boldsymbol {b} \boldsymbol {b}^{\intercal } \boldsymbol {X}_{k}$ big, and this helps the algorithm detect the eigenvector, which is the estimator of the true loading vector.

Second, results of ssmCIA from the scenarios with *n*_*en*_=(5,5,5) show a better performance than those from the scenarios with *n*_*en*_=(3,4,5) and the results from the scenarios with *n*_*el*_=10 show a better performance than those from the scenarios with *n*_*el*_=20 in terms of sensitivity. Again, this agrees with the nature of our generative model. This is because the true loading vectors from the scenarios with *n*_*en*_=(3,4,5) has bigger nonzero valued elements compared to the scenarios with *n*_*en*_=(5,5,5), and the coefficients of connected variables in the network are bigger in the scenarios with *n*_*el*_=10 than those in the scenarios with *n*_*el*_=20.

## Data analysis

### NCI60 dataset

The NCI60 dataset includes a panel of 60 diverse human cancer cell lines used by the Developmental Therapeutics Program (DTP) of the U.S. National Cancer Institute (NCI) to screen over 100,000 chemical compounds and natural products. It consists of 9 cancer types; leukemia, melanomas, ovarian, renal, breast, prostate, colon, lung, and CNS origin. There are various -omics datasets generated from the cell line panel including gene expression datasets from various platforms, protein abundance datasets, and methylation datasets.

The goal of the analysis is to identify a subset of biomarker genes that contributes to the explanation of common relationships among multiple datasets. We downloaded three datasets generated using NCI-60 cell lines from CellMiner [32], two of which were gene expression datasets and the other was protein abundance dataset. Two gene expression datasets were obtained from different technologies, one was the Affymetrix HG-U133 chips [33] and the other was the Agilent Whole Human Genome Oligo Microarray [23]. The third dataset was the proteomics dataset using high-density reverse-phase lysate microarrays [29]. Since one melanoma cell line was not available in the Affymetrix data, We used 59 cell line data that are common to all three datasets. To reduce the computational burden, we selected top 5% of genes with high variance, which resulted in 491 genes in the Affymetrix data, 488 genes in the Agilent data, and 94 proteins in proteomics data. Pathway graph information was obtained from the Kyoto Encyclopedia of Genes and Genomes (KEGG) pathway database [18].

### Analysis results

Table 4 shows the number of nonzero elements in each estimated loading and the percentage of explained data variability by each method. Our sparse methods show comparable or better performance in terms of explained variability with much smaller number of nonzero elements in the estimated loadings. Percentage of explained variability is calculated as a ratio of pseudo eigenvalues corresponding to the estimated loading vectors to the sum of total eigenvalues of the datasets. We applied the estimated loading vectors to the test dataset and the whole dataset to calculate the percentage of explained variability. When we apply the estimated loading to the whole dataset, our sparse methods explain almost the same amount of variability as mCIA with much fewer selected genes. When we apply the estimated loadings to the test dataset, both sparse methods explain comparable amount of variability as mCIA explains using the first estimated loading vector. Moreover, the first two loading vectors of ssmCIA explain more variability than mCIA with much more sparsely estimated loadings.

**Table 4:** For each method, the first two columns show the number of nonzero elements in the first two estimated coefficient loadings of three datasets, the Affymetrix, the Agilent, and the protein dataset respectively. Next four columns contain pseudo-eigenvalues calculated using the estimated coefficient loadings from the training dataset. Last four columns include proportions of pseudo-eigenvalues to the sum of total eigenvalues for each dataset

	# of nonzeros		Pseudo Eigenvalues				% of variability explained			
			test dataset		whole dataset		test dataset		whole dataset	
	1st	2nd	1st	1st + 2nd	1st	1st + 2nd	1st	1st + 2nd	1st	1st + 2nd
mCIA	(491,488,94)	(491,488,94)	36065.92	33447.03	282991.70	218372.50	0.088	0.169	0.129	0.229
smCIA	(250,30,20)	(100,80,15)	31161.89	21283.77	208966.30	157045.80	0.076	0.127	0.095	0.167
ssmCIA	(300,80,15)	(400,15,30)	34611.11	36793.08	239050.80	239050.80	0.084	0.173	0.109	0.218

**Fig. 1:**
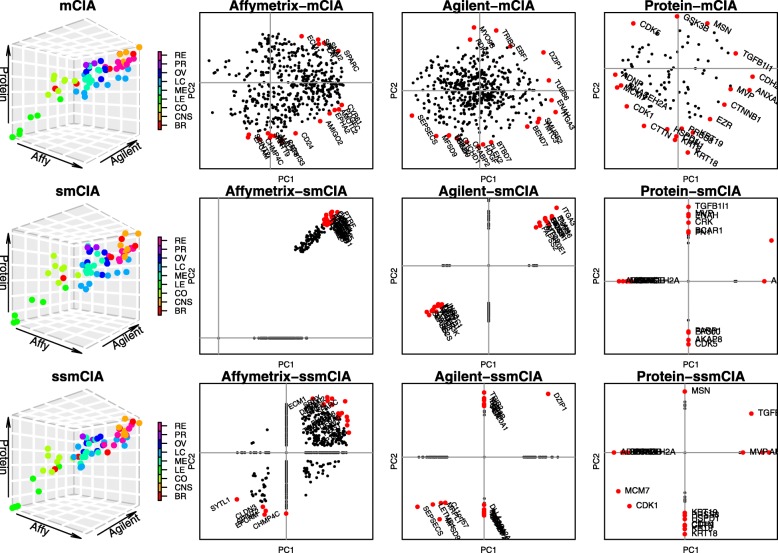
From the top to bottom, each row shows the results from mCIA, smCIA, and ssmCIA method respectively. From left to right, each column represents the sample space in $\mathbb {R}^{n}$, the gene space of the Affymetrix dataset in $\mathbb {R}^{491}$, the gene space of the Agilent dataset in $\mathbb {R}^{488}$, and the gene space of the proteomics dataset in $\mathbb {R}^{94}$. For three panels in the first column, the estimates of the first loading vectors are used. Each different colors represent different cell lines, breast (BR), melanoma (ME), colon (CO), ovarian (OV), renal (RE), lung (LC), central nervous system (CNS, glioblastoma), prostate (PR) cancers and leukemia (LE). For the remaining plots, the estimates of the first two loading vectors are used. Also, colored and labeled points in the plots are top 20 genes that are most distant from the origin, which are more significant compared to other genes. Complete lists of top 20 genes for each panel can be found in the supplementary materials

Four plots generated using the first two estimated loading vectors from each method are shown in Fig. 1. Plots in the first column are 3-D figures where each point represents one cell line sample. The coordinate of each point consists of scores calculated using the first estimated loading vectors of three datasets. Plots from the second to fourth columns are generated using the first two estimated loading vectors on the variable spaces of each data.

Figure 1 proves that sparse estimates from our proposed methods reveal biologically meaningful results that are consistent with previous studies [7, 24]. In the 3-D plot, leukemia cells are well separated from other cells. And we confirmed that smCIA and ssmCIA select certain genes related to leukemia. For example, SPARC is also high weight on both axes of Affymetrix plot from mCIA, smCIA, and ssmCIA analysis. Recent study showed that this gene promotes the growth of leukemic cell [1]. EPCAM is an another example, the gene having a high negative weight on the second axis in the plot of mCIA and ssmCIA in the Affymetrix dataset. This gene is known to be frequently over-expressed in patients with acute myeloid leukemia (AML) [42]. The gene EBF1, another example, has a high weight on the second axis in plot of ssmCIA in the Agilent data, which can be supported by recent studies discussing the relationship between this gene and leukemia [30]. Also, above observations implies that the second axis of the ssmCIA analysis may contribute to cluster the dataset into leukemia cells and non-leukemia cells. From the comparison between the results of smCIA and ssmCIA, we notice that the ssmCIA results is more consistent with the result of mCIA than the results of smCIA, in terms of number of common genes and estimated coefficients of those common genes. Selected genes from ssmCIA has more common genes with mCIA than smCIA. We compared top 30 genes in each datasets and smCIA selected 40 common genes with mCIA while ssmCIA selected 56 genes in common with mCIA. Also, ssmCIA results shows consistent direction for estimated coefficients of genes that are common with the results of mCIA, while some of genes from smCIA shows different directions compared to mCIA results. From this observation, we confirm that incorporation of network information guides the model to achieve the more biologically meaningful estimate results.

In addition, we have conducted a pathway enrichment analysis using ToppGene Suite [5] to assess the set of features selected by our methods. Note that we compare the result using the first estimated loading vectors only. There are numerous gene ontology terms (GO), pathways, and diseases that genes with nonzero values in the estimated loading vectors are enriched. For example, the GO term, regulation of cell proliferation, is revealed to be highly enriched in our results (GO:0042127, Bonferroni adjusted p-values are 5.77*e*^−16^ in the result of smCIA, 7.52*e*^−19^ in the result of ssmCIA). Leukemia-cell proliferation is a topic of interest to researchers [2, 28]. Recently, [11] have reviewed the molecular mechanism related the cell proliferation in leukemia. Also, we confirm that ssmCIA enjoys the benefit of incorporating the network information from the pathway enrichment results. Compared to the results from smCIA, the enrichment results of ssmCIA often shows much smaller Bonferroni adjusted p-values, above GO:0042127 is one of examples. Also, we could obtain more enriched results from ssmCIA than those from smCIA. There are 673 enriched GO terms, pathways, human phenotypes, and diseases in the results of ssmCIA, while 520 enriched results are obtained from smCIA. These results indicate that ssmCIA is more sensitive to select relevant features by incorporating structural information so that more biologically meaningful genes can be identified.

## Discussion

For integrative analysis of *K* data sets, the number of tuning parameters is *K* and 2*K* for smCIA and ssmCIA respectively. As such, the computational costs of the methods can become prohibitively expensive for integrative analysis of a large number of -omics datasets using the proposed cross validation strategy for parameter tuning. One potential solution is to use the same pair of tuning parameter values for all *K* data sets. It is of potential interest to tackle this limitation in future research.

## Conclusion

In this article, we propose smCIA method that imposes a sparsity penalty on mCIA loading vectors and ssmCIA that employs a network-based penalty to incorporate biological information represented by a graph. Our numerical studies demonstrate that both methods are useful for integrative analysis of multiple high-dimensional datasets. Particularly, they yield sparse estimates of the loading vectors while explaining a similar amount of variance of the data compared to the mCIA. In the real data analysis, ssmCIA, with incorporation of biological information, is able to select important pathways contributing to correspondence among the three datasets, and hence yields more interpretable results.

## Data Availability

Our algorithms are implemented by the free statistical software language R and are freely available at: https://www.med.upenn.edu/long-lab/software.html. Three -omics datasets used for the real data analysis can be obtained from the CellMiner webpage https://discover.nci.nih.gov/cellminer/. Additional simulation results and the list of top 30 genes from the NCI60 data analysis can be found in the supplementary materials.

## Abbreviations

mCIA: Multiple co-inertia analysis
smCIA: Sparse multiple co-inertia analysis
ssmCIA: Structured sparse co-inertia analysis
NCI: National cancer institute
CCA: Canonical correlation analysis
Rifle: Truncated Rayleigh flow method
GO: Gene ontology term
